# DICER1 and PRKRA in Colon Adenocarcinoma

**DOI:** 10.4137/bmi.s600

**Published:** 2008-04-28

**Authors:** S. Chiosea, M. Acquafondata, J. Luo, SF. Kuan, RR. Seethala

**Affiliations:** Department of Pathology, University of Pittsburgh Medical Center, Pittsburgh, PA, U.S.A

**Keywords:** DICER1, PRKRA, microRNA machinery, colon adenocarcinoma

## Abstract

Differential microRNA expression in colon adenocarcinoma (CA) was previously reported. MicroRNA biogenesis and function requires a set of proteins designated as the microRNA machinery, which includes DICER1 and PRKRA. Loss of heterozygosity at 14q32.13 *DICER1* locus was detected in up to 60% of CA cases. The *in silico* gene array analysis of CA showed down-regulation of *DICER1* and an up-regulation of *PRKRA*. Immunohistochemically, DICER1 expression was abnormal in 65% of CA (95 of 147 cases). PRKRA was deregulated in 70% of CA (32 of 46 cases). Expression of DICER1 and PRKRA was correlated with clinicopathologic features of CA. DICER1 up-regulation was seen more commonly in women. Only 10 of 46 cases immunostained for both DICER1 and PRKRA showed normal levels of both DICER1 and PRKRA. Microsatellite status of 32 cases was determined. Microsatellite instable cases showed DICER1 up-regulation more commonly when compared to microsatellite stable cases; however, this trend was not statistically significant. Abnormal DICER1 and/or PRKRA expression might explain the observed changes in microRNA profile. The status of the endogenous DICER1 and PRKRA in CA may help to predict the response to future RNA interference-based therapy.

## Introduction

MicroRNAs (miRs) are a class of small noncoding 21-nt long RNAs that have been implicated in the development of multiple types of human malignancies (Zhang, 2006), including colon adenocarcinoma (CA) (Bandres, 2006; Lanza, 2007; Michael, 2003; Volinia, 2006). Production and function of miR requires a set of proteins designated as the miR machinery (summarized in (Chiosea, 2006)). In the cytoplasm, DICER1 cuts both strands of the pre-miR duplex, generating a mature ~21-nucleotide miR duplex (Hutvagner, 2001). PRKRA (protein kinase, interferon-inducible double-stranded RNA-dependent activator) has been shown to interact with DICER1 without facilitating its pre-miRNA cleavage activity (Lee, 2006).

In a miR-guided fashion, the miR machinery regulates the expression of multiple tumor suppressor genes and oncogenes (Hammond, 2006). The list of miR with known cancer gene targets continues to grow (e.g. bcl-2, c-myc, RAS) (Calin and Croce, 2006). Independently of the RNAi pathway, DICER1 controls checkpoints in response to mutagenic stress (Carmichael, 2004).

Mature miR-143 and miR-145 exhibit reduced levels in neoplastic colorectal tissue. This occurs in the background of constant levels of miR-143 and miR-145 precursors in both normal and tumor tissues (Michael, 2003). Recently, significant differences were described in miRNA expression between microsatellite stable and microsatellite instable CA (Lanza, 2007). In part, this and other changes in miR expression could be due to altered levels of microRNA-associated proteins. For instance, the overall incidence of loss of heterozygosity at *DICER1* locus is 60% in the distal colon and 28.6% in the proximal colon (Bando, 1999). Here, we present the *in silico* transcriptional analysis of *DICER1* and *PRKRA* and further describe alterations of DICER1 and PRKRA expression in CA.

## Material and Methods

### Clinical profile of cases and screening paraffin-embedded tissue microarray (TMA)

The demographic and clinicopathologic features of the patients in this study are listed in Table 1. TMAs were constructed using a semi-automatic tissue arrayer (Chemicon, San Diego, CA). Formalin-fixed paraffin-embedded tumor blocks were selected and marked after microscopic examination of corresponding hematoxylin and eosin stained slides. One-millimeter cores were extracted from marked areas and arrayed in duplicate onto two recipient blocks. To assess the potentially different DICER1 expression at the invasive front of cancer, whole sections of eight cases included in TMA and 20 additional cases were stained for DICER1.

### Immunohistochemical stains and statistical analysis

Immunohistochemistry was performed as previously described (Chiosea, 2006). Briefly, the slides were incubated at 4 °C overnight with anti-DICER1 antibodies (Clonegene, Hartford, CT, 1:400 dilution) and anti-PRKRA (Santa Cruz Biotechnology, Santa Cruz, CA, 1:800). The non-neoplastic colonic mucosa epithelium adjacent to the areas of neoplasm served as an internal positive control. The intensity for the DICER1 and PRKRA was graded semiquantitatively on a scale from 1 to 3. The staining was scored as “1” if the staining intensity in the neoplasm was lower than the staining intensity of the normal colonic epithelium. The staining was scored as “2” if the staining intensity in the neoplasm matched the staining intensity of the normal colonic epithelium. The staining was scored as “3” if the staining intensity in the neoplasms exceeded the staining intensity of the normal colonic. Two pathologists (R.S. and S.C.) scored the stained slides. The final score assigned to the case was the average of the two cores rounded up to the nearest numeric category. For instance, when the average score after analyzing 2 or more cores from one case was 1.5 it was counted as less than 2, i.e. a score of “1” was assigned. Statistical analysis was performed with SPSS 14.0 software (SPSS Inc, Chicago, IL). Two group comparisons were performed with the Fisher exact test with a p-value of <0.05 considered statistically significant.

### Microsatellite status

was determined as previously described (Trusky, 2006). To select cases for microsatellite status evaluation modified Bethesda criteria were applied to consecutive cases of CA (outlined in (Gologan, 2005)). Shortly, under direct visualization using a stereomicroscope normal tissue and tumor targets from 6 unstained sequential blank slides at 5 μm thick and radius of at least 6 mm were microdissected. Dissection was performed according to the marked H and E slides. The tumor targets are selected for >90% tumor cells. Areas of stroma and necrosis were avoided. The samples were treated with proteinase K (New England Biolabs, Ipswich, MA) overnight at 60 °C and then DNA was extracted using the Qiagen DNEasy kit (Qiagen, Valencia, CA) according to manufacture’s recommendations. DNA concentration was obtained for each sample by using a spectrophotometer (Nanodrop Technologies, Wilmington, DE). The 260/280 ratio was calculated to confirm purity in the samples. PCR was then performed using 1 mL of purified DNA and fluorescently labeled primers designed for the 5 standard NCI-recommended microsatellite repeat loci (BAT25, BAT26, D2S123, D5S346, and D17S250), 2 mono-nucleotides, and 3 dinucleotides (Boland, 1998). PCR products were semiquantitatively analyzed using the ABI Prism 3100 and Genescan software (Applied Biosystems, Foster City, CA). Interpretation was performed as previously described (Trusky, 2006).

## Results

### *In Silico* analysis of expression array data reveals deregulation of *DICER1* and *PRKRA* in CA

**Figure 1:**
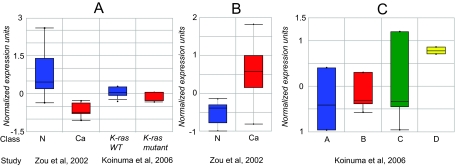
Oncomine meta-analysis of CA expression arrays for miR machinery-associated genes. The box plot is the interquartile range and the whiskers are the 10–90th% range. Normalized expression units are log2 transformed. Array Median is set to 0 and array standard deviation is set to 1. **A.** *DICER1* was significantly down-regulated in CA samples (n = 9) when compared to normal colonic mucosa samples (n = 8). *DICER1* was down-regulated in CA specimens positive for *K-Ras* mutation (n = 8) when compared to *K-Ras* wild type samples (n = 32). **B.** *Ago2* was significantly up-regulated in CA. **C.** CA at an advanced stage (Duke D, n = 6) showed higher *PRKRA* level than earlier stages of CA (Duke A, n = 6; Duke B, n = 14, and Duke C, n = 16).

Using Oncomine, a newly developed, Internet-based query tool (www.Oncomine.org) (Rhodes, 2004), we were able to query 5 previously published expression array datasets of CA for *DICER1* and *PRKRA*. These data althouth publicly available for analysis through Oncomine, were never presented before. All studies had significance level of P < 0.05. The *DICER1* expression was altered in 2 of 5 studies. *DICER1* was down regulated in CA compared to normal colon (Table 2 and Fig. 1A). Also, lower *DICER1* level was seen in CA with *K-ras* mutation when compared to wild type K*-ras* (Table 3 and Fig. 1A) (Koinuma, 2006; Zou, 2002).

Equal *PRKRA* expression characterizes normal colon tissue and CA (Table 2). However, CA at advanced stages showed higher *PRKRA* level than earlier stages of CA (−0.4 normalized expression units in Duke Stage A vs. 0.8 in Duke Stage D) (Table 2 and Fig. 1C. *PRKRA* was up-regulated in CA with wild type *BRAF* (Table 3)(Koinuma, 2006).

Changes of other miR machinery genes are summarized in Tables 2 and 3.

### Immunohistochemical analysis of DICER1 and PRKRA expression in CA

**Figure 2:**
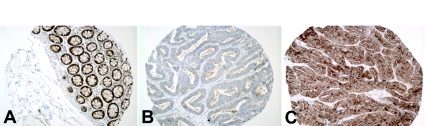
DICER1 immunoreactivity in normal colon mucosa and CA. **A.** Normal colonic mucosa. DICER1 immunoreactivity is limited to the basal rim of cytoplasm. **B.** A representative example of CA with loss of DICER1. **C.** A representative example of CA with increased diffuse cytoplasmic DICER1 expression. Immunohistochemistry, ×200 original magnification.

DICER1 and PRKRA expression in normal colon was limited to mucosal epithelium (Fig. 2). Both proteins showed similar cytoplasmic expression. One hundred forty seven cases of CA were stained for DICER1. DICER1 expression was abnormal in 65% (95 of 147) tumors. Fifty-four cases of CA showed up-regulation, 43 CA showed down-regulation and 52 cases had normal immunoreactivity for DICER1. DICER1 was more commonly down-regulated among women (p = 0.05). In whole sections, there was minor regional heterogeneity but no predilection for tumoral regions (i.e. surface component, etc. invasive front).

Forty-one cases were stained for PRKRA, also. PRKRA was deregulated in CA in 70% (32 of 46 cases). PRKRA level was increased in 43% of CA (20 of 46 cases) and decreased in 26% of CA (12 of 46 cases). Expression of DICER1 and PRKRA is summarized in Table 4. Expression of DICER1 or PRKRA did not correlate with TNM staging parameters, independently or by stage group, laterality, and age. Only 10 cases showed normal levels of both DICER1 and PRKRA.

To address the question of DICER1 expression and mismatch repair system we analyzed DICER1 expression in 15 cases of mcirosatellite stable (MSS) CA and 18 cases of microsatellite instable (MSI) CA (including 5 cases of hereditary nonpolyposis colorectal carcinoma syndrome, HNPCC). Most MSI cases showed DICER1 up-regulation (10 of 18 cases); however, this trend is not statistically significant when compared to MSS cases. Normal DICER1 immunoreactivity was seen in 5 of 18 MSI cases.

## Discussion

Differential miR expression in CA was shown by several independent studies (Bandres, 2006; Michael, 2003; Volinia, 2006). The suggested set of miRs differentiating normal from cancer tissues is composed of 18 miRNA, 10 down-regulated and 8 up-regulated (Bandres, 2006). In a recent analysis of 23 microsatellite stabile (MSS), CA and 16 CA with microsatellite instability (MSI), 8 microRNAs were up-regulated and, along with 15 mRNA, could correctly distinguish MSI versus MSS CA samples (Lanza, 2007).

Little is known about the mechanisms of miR regulation in normal and neoplastic tissues. In a non-cancer type specific study, miR genes were found to be commonly located at minimal regions of amplification, loss of heterozygosity, and breakpoint regions suggesting that abnormal miR profiles can be caused by somatic genetic mutation (Zhang, 2006). However, in colon tumors miR expression did not correlate with the miR gene copy numbers (Lamy, 2006).

New details about the transcriptional and post-transcriptional regulation of miR expression have been discovered: deacetylation of chromatin proteins was thought to be an important factor capable to alter global miR levels (Scott, 2006).

More importantly, tissue-specific miR processing can be regulated by DICER1 (Obernosterer, 2006). Mature miR-143 and miR-145 are reduced in neoplastic colon tissue, while unprocessed precursors of these miRs remain at a constant level (Michael, 2003). This result suggests that transcriptional changes are not accountable for reduced miR levels. More likely reduction in miRs is secondary to the decrease in DICER1-processing activity. Intuitively, reduced DICER1 activity should result in more generalized effect on miR expression. However, in adenocarcinoma cell lines displaying hypomorphic DICER1 phenotype, of 97 known miRs, only 55 were differentially expressed (Cummins, 2006). This observation suggests that DICER1 is required for the biogenesis of only a **subset** of known miRs (Cummins, 2006).

One of five gene array studies in presented *in silico* gene array analysis of CA showed lower *DICER1* level in 9 analyzed CA when compared to normal colon mucosa (Rhodes, 2004; Zou, 2002). We demonstrate that in increased number of specimens an almost equal proportion of CA demonstrates loss or decrease of DICER1 expression. Our results show no correlation between level of DICER1 expression and CA progression. This result is in agreement with the work by Koinuma et al. (Koinuma, 2006). *PRKRA* increase in advanced CA stages shown in the same study is probably quantitatively too minor to be translated into a significant change at PRKRA protein level (Koinuma, 2006). The gene expression data and the protein expression values are derived from different samples and could show conflicting features. However, our samples as well as the samples in Koinuma et al. (2006) are not believed to represent special groups or subtypes of colon tumors, and general features should thus be conserved between the specimens. A limitation of this study is the lack of knowledge on *BRAF* and *K-Ras* status of cases analyzed for DICER1immunoreactivity.

It has been shown that the overall incidence of LOH at 14q32 *DICER1* locus (D14S267 marker) in the distal large bowel (60%, 21/35) tended to be significantly higher than that in the proximal bowel (28.6%, 4/14) (Bando, 1999). Our more extended study did not show correlation between DICER1 expression and the site of CA.

We have previously demonstrated the role of DICER1 in the development of prostate adenocarcinoma (Chiosea, 2006) and non-small cell lung carcinoma (Chiosea, 2007). DICER1 expression varies in squamous cell carcinoma of the esophagus and does not show significant association with patient survival (Sugito, 2006). Together with DICER1 and PRKRA changes in CA described here, alterations of microRNA machienry might be a new common theme of carcinogenesis.

Interestingly, we found decreased expression of DICER1 in colon tumors from females. miR machinery may be one of probably several biomarkers differentially expressed across gender. One epidemiologic study suggests that women present with more aggressive disease when controlled for age, race and anatomic subsite (Woods, 2005). However, the review of the Surveillance, Epidemiology, and End Results Program database yielded the opposite results (Wingo, 1998). The contributory role of miR machinery to gender difference has to be validated in a large study.

There is an increasing evidence that potential RNA interference based therapy can have non-specific effects, including off-target silencing and activation of the interferon system (Jackson and Linsley, 2004). Furthermore, as was shown by Grimm et al., shRNA expression in hepatocytes following intravenous infusion can result in fatal dose-dependent liver tissue injury. Adverse effects were most likely caused by competition of exogenous siRNA pathway with the endogenous miR pathway, for instance Exportin-5 (Grimm, 2006). The widespread changes of DICER1 and PRKRA expression in CA described here, might not only explain some abnormalities in the miR profile of CA, but also may help to predict the response to future RNAi-based therapy.

## Figures and Tables

**Table 1:** Clinicopathologic and demographic characterization of the patients.

Mean age, years (range)		68 (24 to 93)
>50		129
<50		18
Gender	Male	61
	Female	86
Normal colon tissue		11
Colorectal carcinoma		147
Laterality	Right	79
	Left	68
T stage		
In situ		1
T1		13
T2		24
T3		101
T4		8
Lymph node status, N0		74
Lymph node status, N1 (1–3)		40
Lymph node status, N2 (>4)		30
Known distant metastasis		19
MSS		15
MSI (HNPCC)		13 (5)

Cases with both cores missing on all examined TMA sections were excluded from the analysis. Nodal status is unknown in 3 cases. MSS, microsatellite stable; MSI, microsatellite instable.

**Table 2:** MicroRNA machinery changes and colon cancer progression: a summary of gene array studies.

MicroRNA machinery-associated genes, median, normalized expression units												
*DICER1*	0.5	−0.7										
*EIF2C2*	−0.4	0.6	0.2	0.02	0.08	0.3						
*PRKRA*			−0.4	−0.3	−0.3	0.8						
*HSP90*							−0.2	0.2	−0.4	0.2	−0.2	0.3
*EIF2C1*			0.4	0.45	0.4	0.3						
*SND1*							−0.25	0.13	−0.3	0.2	−0.4	0.3
Sample, n	N	CA	A	B	C	D	N	CA	N	CA	N	CA
	8	9	6	14	14	6	12	18	22	40	18	18
Study	(Zou, 2002)		(Koinuma, 2006)				(Graudens, 2006)		(Alon, 1999)		(Notterman, 2001)	

**Abbreviations:** n: number of samples; N: normal colonic mucosa; A, B, C, and D: Duke stages; CA: colon adenocarcinoma.

**Table 3:** MicroRNA machinery genes change with *BRAF, K-ras* mutations and Microsatellite Instability (MSI) status of CA.

MicroRNA machinery-associated gene, median, normalized expression units						
*DICER1*					0.05	−0.3
*EIF2C2*	0.4	−0.1	0.4	−0.05		
*PRKRA*	0.6	−0.4				
*TNRC6B*	−0.18	0.17	−0.11	0.16	0.02	−0.27
*SND1*	0.3	−0.5	0.4	−0.5	−0.02	0.15
*DDX20*			−0.08	0.1		
Sample, n	WT *BRAF,* 22	Mutant *BRAF,* 18	MSI Neg, 20	MSI Pos, 20	K-Ras WT, 32	Mutant *K-Ras,* 8
Study	(Koinuma, 2006)					

**Table 4:** Expression of both DICER1 and PRKRA in CA, number of cases.

PRKRA/DICER1	Up	N	Down
Up	9	6	5
N	2	5	7
Down	2	4	6
